# Morphology Control and Mechanism of Different Bath Systems in Cu/SiCw Composite Electroplating

**DOI:** 10.3390/nano14121043

**Published:** 2024-06-18

**Authors:** Bing Niu, Dongdong Xie, Yanxin Zhang, Yuxiao Bi, Yigui Li, Guifu Ding, Liyan Lai

**Affiliations:** 1 School of Science, Shanghai Institute of Technology, Shanghai 201418, China; bingniu1552@163.com (B.N.); biyuxiao1999@163.com (Y.B.); ygli@sit.edu.cn (Y.L.); 2 National Key Laboratory of Science and Technology on Micro/Nano Fabrication, School of Electronic Information and Electrical Engineering, Shanghai Jiao Tong University, Shanghai 200240, China; xiedongdong@sjtu.edu.cn (D.X.); zhangyx2019@sjtu.edu.cn (Y.Z.); gfding@sjtu.edu.cn (G.D.)

**Keywords:** thermal management materials, composite electrodeposition, microstructure, SiCw toughened

## Abstract

With the rapid development of electronic technology and large-scale integrated circuit devices, it is very important to develop thermal management materials with high thermal conductivity. Silicon carbide whisker-reinforced copper matrix (Cu/SiCw) composites are considered to be one of the best candidates for future electronic device radiators. However, at present, most of these materials are produced by high-temperature and high-pressure processes, which are expensive and prone to interfacial reactions. To explore the plating solution system suitable for SiCw and Cu composite electroplating, we tried two different Cu-based plating solutions, namely a Systek UVF 100 plating solution of the copper sulfate (CuSO_4_) system and a Through Silicon Via (TSV) plating solution of the copper methanesulfonate (Cu(CH_3_SO_3_)_2_) system. In this paper, Cu/SiCw composites were prepared by composite electrodeposition. The morphology of the coating under two different plating liquid systems was compared, and the mechanism of formation of the different morphologies was analyzed. The results show that when the concentration of SiCw in the bath is 1.2 g/L, the surface of the Cu/SiCw composite coating prepared by the CuSO_4_ bath has more whiskers with irregular distribution and the coating is very smooth, but there are pores at the junction of the whiskers and Cu. There are a large number of irregularly distributed whiskers on the surface of the Cu/SiCw composite coating prepared with the copper methanesulfonate (Cu(CH_3_SO_3_)_2_) system. The surface of the composite is flat, and Cu grows along the whisker structure. The whisker and Cu form a good combination, and there is no pore in the cross-section of the coating. The observation at the cross-section also reveals some characteristics of the toughening mechanism of SiCw, including crack deflection, bridging and whisker pull-out. The existence of these mechanisms indicates that SiCw plays a toughening role in the composites. A suitable plating solution system was selected for the preparation of high-performance Cu/SiCw thermal management materials with the composite electrodeposition process.

## 1. Introduction

With the rapid development of electronic technology and large-scale integrated circuit devices, it is very important to dissipate the heat generated by high-power electronic components in the working process. At present, there are two main ways to improve the cooling capacity of chips [1]: first, the development of high-thermal-conductivity thermal management materials to improve the heat transfer and heat dissipation capacity of the package structure; second, the development of a new heat dissipation mechanism to improve cooling efficiency. Among them, the former is one of the supporting elements of the latter, and reducing the temperature rise in the chip by selecting appropriate heat sink materials is the most commonly used method for the thermal management of electronic systems. Thermal management materials are a general term for packaging materials with good thermal properties. Their cores contain high-performance thermal sink materials, and their key characteristics include high thermal conductivity (TC) and low coefficient of thermal expansion (CTE). With the increase in chip heating intensity and continuous innovation, research on new heat sink materials has been widely valued [2,3,4,5,6].

The first generation of heat sink materials was all-metal materials, mainly Kovar (iron–nickel–cobalt alloy) and Cu/W, Cu/Mo composite materials [7]. With the main goal of reducing the CTE of metal materials, the technology has since matured, but there are still a lot of applications in ordinary power chip packaging. Furthermore, their thermal conductivity is not high enough, and their CTE is not fully matched. Therefore, various new heat sink materials are constantly emerging, and the requirements are also increasing. At present, the ideal heat sink material should have at least four characteristics: high thermal conductivity, low thermal expansion coefficient, superior micro-machining and good mechanical strength [8]. Compared with the standard of ideal heat sink materials, metal matrix composites (MMCs) composed of high-thermal-conductivity matrix metals and reinforcements have the most promising development potential. Among them, silicon aluminum carbide (AlSiC) is the first to achieve success because of its good balance between thermal conductivity and thermal expansion coefficient. Although it does not fully meet all the expectations of ideal heat sink materials, it shows the attractive development potential of this kind of material. Drawing on AlSiC development ideas, people have focused their attention on copper-based silicon carbide composites (Cu/SiC). Because Cu has many comparative advantages compared with Al, it is expected that Cu/SiC should have higher thermal conductivity and a lower thermal expansion coefficient [9].

In order to study the new generation of heat sink materials, a lot of research has been conducted. Shan et al. [10] prepared Cu/SiCp electronic packaging materials with different volume fractions using hot pressing sintering technology and studied the relative density, thermal expansion and thermal conductivity of the composites. Cai et al. [11] prepared Cu/SiC composites with different mass fractions by vacuum hot pressing and studied the effect of SiC content on their mechanical properties. Feng et al. [12] prepared Cu/SiC composites with different volume fractions using powder metallurgy and hot extrusion.

However, the thermal conductivity of Cu/SiC composite heat sink materials prepared by various synthesis methods is not as high as expected. It is found that the dissolution of Si into the copper matrix in the high-temperature preparation process is the fundamental reason for the significant decrease in thermal conductivity [13,14,15]. Therefore, in order to prevent the reaction of Cu and SiC at high temperatures, many attempts have been made, among which passivation protection of the reinforcing phase has become the best choice. Th. Schubert et al. [16] coated a layer of Mo film on the surface of SiCp by vapor deposition and formed an interface barrier layer using the reaction of Mo and SiC to form molybdenum carbides and silicides. Z. Xu et al. [17] chose titanium plating on SiCw to slow down the interfacial reaction between SiCw and Cu. It has been found that even if the passivation layer is used to protect the reinforcing phase, the effect is not significant [18]. The interfacial reaction between Cu and SiC at high temperatures has become an insurmountable obstacle to the development of Cu/SiCp heat sink materials. Therefore, it is imperative to carry out innovative research on low-temperature processes. In addition, most of the current research on Cu/SiC composites uses silicon carbide particles as the reinforcing phase, which is not helpful to the mechanical strength of the composites, as well as crystalline silicon carbide powder, but silicon carbide whisker (SiCw) is a more advantageous choice. Compared with SiCp, SiCw has a higher thermal conductivity and a larger aspect ratio. If it can be used as a reinforcing phase to prepare Cu/SiCw composite heat sink materials based on low-temperature processes, it will surpass the thermal conductivity and mechanical strength simultaneously; fully meet the four standards of ideal heat sink materials; and have many potential technical advantages such as low cost, easy availability and environmental stability.

Composite electrodeposition technology is a process in which nanoparticles are embedded in metal coatings to co-deposit nanoparticles with metal ions. The unique physical and chemical properties of nanoparticles are given to metal coatings. This method does not need to consider interface reactions. The process equipment is simple, the scope of application is wide and the electrodeposition technology is compatible with microelectronic devices. It is thus considered to be a very promising technology. Lai et al. [19] successfully prepared Ni/SiCw composites using composite electrodeposition technology and characterized the composites with XPS [20] and XRD [21] methods. The results show that no impurities are formed at the interface between the whisker and Ni, and the addition of a whisker has a significant effect on the mechanical properties of the composite coating. When the content of SiCw in the plating solution was 0.8 g/L, the tensile strength of the composite coating was increased by 2.6 times, and the elastic modulus was increased by 42%. Given this, in this study, Cu/SiCw composites were prepared by composite electrodeposition, the morphology of the coating under two different plating liquid systems was compared and the mechanism of formation of the different morphologies was analyzed. The results show that SiCw is well combined with Cu in the TSV bath of the Cu(CH_3_SO_3_)_2_ system, and bottom-up void-free filling is achieved. Under the Systek UVF 100 plating solution of the CuSO_4_ system, SiCw combines poorly with Cu, and there are more pores in the composite.

## 2. Experiments

### 2.1. Materials

**Figure 1:**
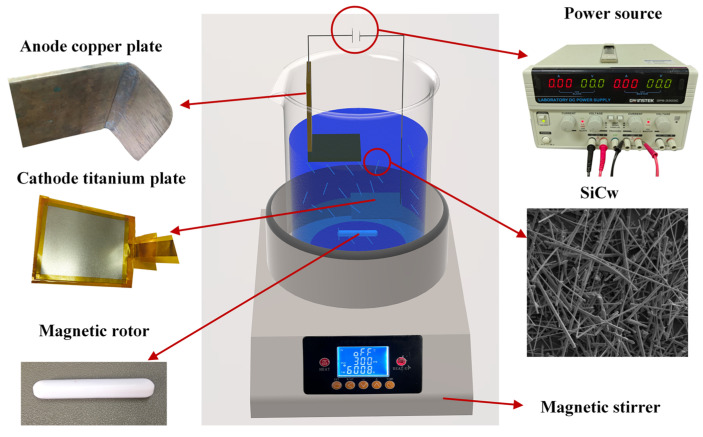
Preparation process of Cu/SiCw composites.

Cu/SiCw composites were prepared by composite electrodeposition technology. The effects of two different plating liquid systems with copper methanesulfonate and copper sulfate as the main salts on the coating were studied. The compositions of the plating solution are shown in Table 1 and Table 2. The TSV plating solution additive system (DVF-B, DVF-C and DVF-D) is a commercial additive based on Enthone Inc., Piscataway, NJ, USA (MICROFAB DVF 200), and the Systek UVF 100 plating solution additive system (SYSTEK UVF 8470, SYSTEK UVF 8471 and SYSTEK UVF 8472) is a commercial additive based on MacDermid Alpha, Piscataway, NJ, USA (Systek UVF 100). An electroplating schematic diagram is shown in Figure 1. The anode size of the phosphor copper plate was 40 mm × 50 mm × 3 mm, the cathode used a 40 mm × 50 mm titanium sheet material, and the selected whisker was commercial β-SiCw (XFNANO Material Co., Ltd., Nanjing, China) with a length of 10~50 μm and a diameter of 300~500 nm. Since the density of SiCw (3.216 g/cm^2^) is greater than the density of the plating solution (1.32 g/cm^2^), in order to avoid the self-sinking of SiCw affected by gravity and to evenly distribute SiCw in the coating, this experiment adopts the method of parallel placement of the electrode with the bottom of the beaker, and the anode is above the cathode.

### 2.2. Preparation of Cu/SiCw Composites

Before electroplating, SiCw was pre-cleaned to remove surface impurities. SiCw was added to hydrochloric acid (60 mol/L) for ultrasonic treatment for 30 min and rinsed with deionized water until it reached a neutral pH. Then, SiCw was added to a NaOH solution (10% wt) for ultrasonic treatment for 30 min, rinsed with deionized water until it reached a neutral pH and dried for later use. In order to obtain the best electroplating conditions and to prepare composites with excellent properties, a series of studies on the preparation of diamond-reinforced Cu matrix composites using electrodeposition, conducted by Liyan Lai’s team, were referenced [22,23,24,25]. After the Cu/SiCw condition optimization test, the composite coating was prepared under the following excellent process conditions: 25 °C, 300 rpm and 10 mA/cm^2^ [22,23]. After the electrodeposition, the composite coating was ultrasonically cleaned in deionized water for 10 min to remove any loosely adsorbed SiCw and the residual plating solution from the surface of the coating. In order to prevent oxidation of the copper surface, a vacuum oven was used for baking at 50 °C for 10 min to remove the water on the surface of the coating.

### 2.3. Characterization

The surface morphology and cross-sectional morphology of the SiCw/Cu composites were characterized using a JSM-7800 field emission scanning electron microscope (SEM, ZEISS, Jena, Germany) with an operating voltage of 15 kV. In addition, the distribution of elements was obtained based on the associated energy dispersion spectra (EDS, WITEC, Ulm, Germany). In order to further study the interface behavior of the Cu-SiCw composites, the samples were thinned by a Gatan 695 ion beam reducer, and the interface between copper and SiCw was characterized by transmission electron microscopy (TEM, FEI Talos F200 X, Hillsboro, OR, USA).

## 3. Results and Discussion

### 3.1. Systek UVF 100 Plating Solution

#### 3.1.1. Effect of SiCw Content in Systek UVF 100 Bath on Morphology of Composite Coating

**Figure 2:**
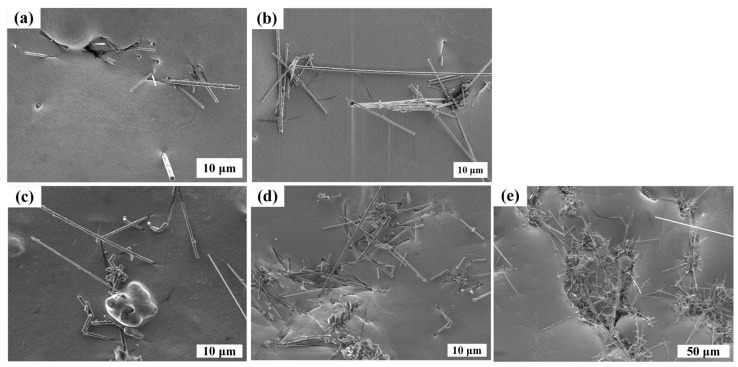
The surface morphology of Cu/SiCw composites with different SiCw contents in the plating solution: (**a**) 0.2 g/L, (**b**) 0.5 g/L, (**c**) 0.8 g/L, (**d**) 1.2 g/L and (**e**) 1.5 g/L.

**Figure 3:**
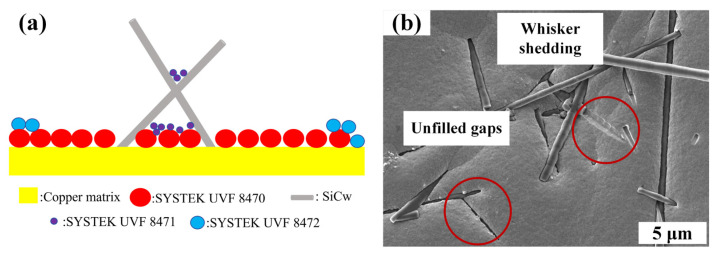
(**a**) Distribution of additives; (**b**) bonding of Cu and SiCw.

In order to obtain high-performance Cu/SiCw composites, different concentrations of SiCw (0.2~1.5 g/L) were added to the Systek UVF 100 plating solution of the CuSO_4_ system under the following optimal process conditions: 25 °C, 300 rpm and 10 mA/cm^2^. The microstructure of Cu/SiCw composites with different contents is shown in Figure 2. As the concentration of SiCw in the plating solution increases, the number of whiskers observed on the surface of the coating increases, which also means that more and more whiskers are embedded into the composite. In addition, the distribution behavior of SiCw at different contents is also different. When the content of SiCw in the plating solution is low, SiCw is randomly distributed in the composite. When the content of SiCw in the plating solution is 1.2 g/L, as shown in Figure 2d, there are a large number of randomly distributed whiskers on the surface of the coating, and the surface of the composite is very flat. The flatness of the coating is mainly attributed to the additives. The distribution of the additives on the cathode surface is shown in Figure 3a. SYSTEK UVF 8470 is usually adsorbed on the cathode surface and interacts with chloride ions to form a thin layer, which reduces the deposition rate of Cu^2+^ by increasing the effective thickness of the diffusion layer. SYSTEK UVF 8471 acts on the bottom of the pores, so that SYSTEK UVF 8470 deposited at the bottom of the pores loses its inhibitory effect and accelerates the filling of the pores. SYSTEK UVF 8472 selectively adsorbs at high-current-density positions such as edges, corners and local protrusions to prevent Cu^2+^ deposition in high-current-density regions. Under the combined action of the three additives, the coating surface is very flat [26,27,28]. When the content of SiCw reaches 1.5 g/L, SiCw agglomerates in the composites, which is not conducive to the properties of the composites. However, because SiCw is a nano-scale material with high surface energy, agglomeration is inevitable with the increase in whisker concentration [29].

In order to further observe the combination of SiCw and Cu on the surface of the coating, the surface of the coating was observed under a high-rate scanning electron microscope. As shown in Figure 3b, the traces of SiCw shedding from the coating after ultrasonic vibration treatment can be clearly observed, which shows the mechanical combination of Cu and whiskers-only landfill. If the composite material receives an external force, the joint will first break. In addition, the surface of the coating also found unfilled gaps after SiCw was completely embedded and pores formed at the interface between whiskers and Cu, which will reduce the mechanical and thermal properties of the composite.

#### 3.1.2. Cross-Sectional Morphology of Systek UVF 100 Composite Coating

**Figure 4:**
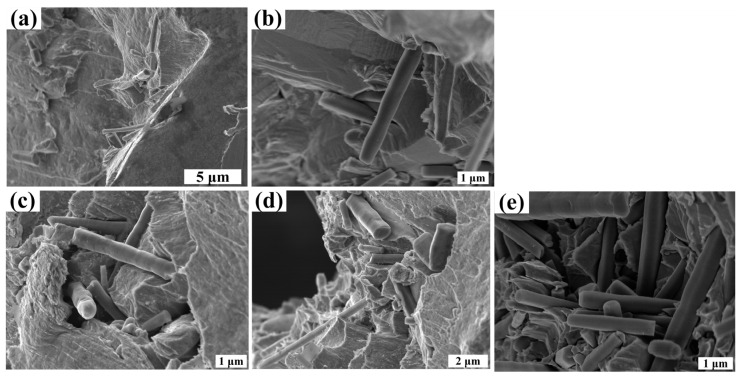
SEM images of the fracture surface of Cu/SiCw composites at different SiCw concentrations in the plating solution: (**a**) 0.2 g/L, (**b**) 0.5 g/L, (**c**) 0.8 g/L, (**d**) 1.2 g /L and (**e**) 1.5 g/L.

In order to further verify whether the interior of the coating is consistent with the surface morphology, we characterized the cross-sectional morphology of the coating. The SEM micrographs of the cross-section of the fracture samples with different SiCw fractions are shown in Figure 4. The fracture surface is uneven, indicating that the crack is not diffused along a plane. The addition of whiskers increases the crack path and consumes more fracture energy. When the concentration of SiCw in the plating solution is 0.2 g/L, as shown in Figure 4a, the whiskers observed at the cross-section are less, and the whiskers at the cross-section can be clearly observed to be pulled out. When the concentration of SiCw in the plating solution is 0.5~1.2 g/L, as shown in Figure 4b–d, the increase in the number of SiCw dispersed in the Cu matrix can be clearly observed on the fracture surface, and more and more whiskers are observed on the fracture surface. Pulling out from the matrix indicates that the consumption of fracture energy is greater, when the content of SiCw increases to 1.5 g/L, as shown in Figure 4e; SiCw agglomerates at the cross-section; and it is difficult for Cu to deposit in the pores at the whisker aggregation to completely fill the pores. At this time, the whisker does not play the role of toughening but weakens the performance of Cu itself, and the aggregated SiCw will lead to stress concentration.

### 3.2. TSV Plating Solution

#### 3.2.1. Effect of SiCw Content in TSV Bath on Morphology of Composite Coating

**Figure 5:**
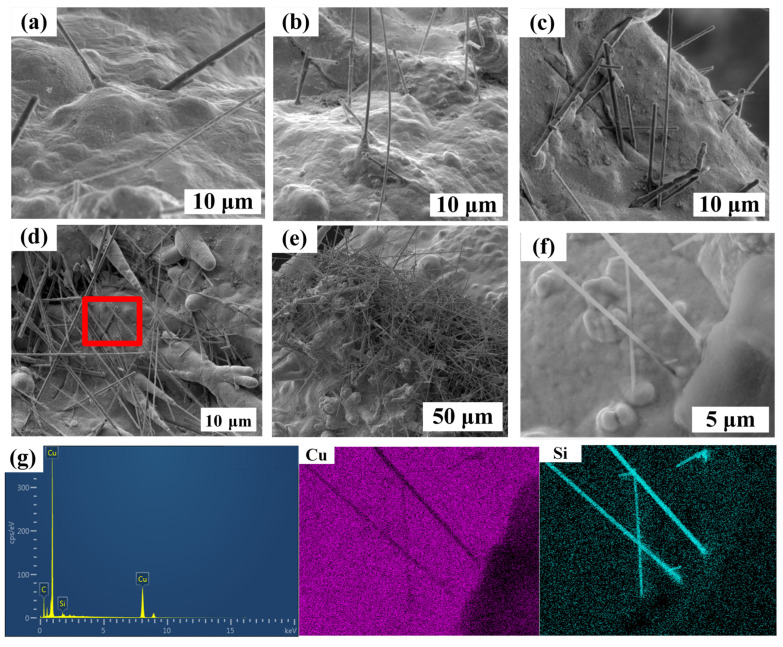
The surface morphology of Cu/SiCw composites with different SiCw contents in the plating solution, (**a**) 0.2 g/L, (**b**) 0.5 g/L, (**c**) 0.8 g/L, (**d**) 1.2 g/L and (**e**) 1.5 g/L, (**d**,**f**) in the enlarged image; (**g**) EDS determination of (**f**) and the corresponding EDS diagram of Si and Cu.

**Figure 6:**
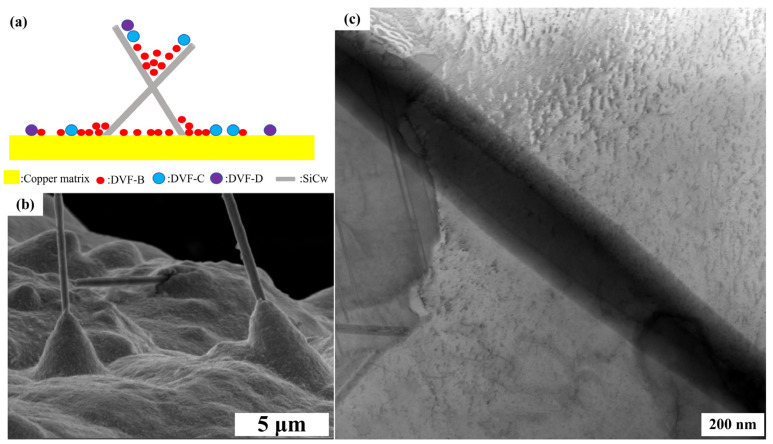
(**a**) Distribution of additives between SiCw, (**b**) bonding of Cu and SiCw, and (**c**) TEM image of Cu/SiCw composites.

In order to obtain high-performance Cu/SiCw composites, different concentrations of SiCw (0.2~1.5 g/L) were added to the TSV plating solution of the Cu(CH_3_SO_3_)_2_ system under the following optimal process conditions: 25 °C, 300 rpm and 10 mA/cm^2^. The microstructure of Cu/SiCw composites with different contents of SiCw is shown in Figure 5. With the increase in the concentration of SiCw in the plating solution, the number of whiskers observed on the surface of the coating also increased, which also means that more and more whiskers are embedded in the composite. In addition, the distribution behavior of SiCw is different at different concentrations. When the SiCw content in the plating solution is low, as shown in Figure 5a–d, SiCw is randomly distributed on the surface of the composite material. When the SiCw content in the plating solution is 1.2 g/L, as shown in Figure 5d, a large number of irregularly distributed whiskers are observed on the surface of the coating, and the surface of the composite material is smooth. The flat coating is attributed to the combined effect of additives. The distribution of additives on the cathode surface is shown in Figure 6a. The diffusion coefficient of DVF-B is large and preferentially diffuses to the bottom of the pores. When the additive DVF-B is adsorbed at the bottom of the pores, it is beneficial to accelerate the crystal growth of copper at the bottom of the pores during copper deposition and prevent the formation of pores in the coating. The diffusion coefficients of DVF-C and DVF-D are small. DVF-C is mainly adsorbed on the upper end of the pore and inhibits the growth of copper at the opening of the pore. DVF-D is a nitrogen-containing organic macromolecule with a positive charge, which is easy to gather in a high potential area. Therefore, it is mainly adsorbed around the coating at the opening of the pore to inhibit the formation of copper nodules [30,31,32,33,34,35]. When the SiCw content reaches 1.5 g/L, as shown in Figure 5e, SiCw also agglomerates on the surface of the composite. In order to further study the morphology of the composite material and observe the interface bonding between Cu and SiCw in the composite material, a typical microscopic study was carried out on the composite material with a SiCw concentration of 1.2 g/L in the plating solution. As shown in Figure 5f, the surface morphology is uniform and dense without pores. The contents of C, Si and Cu were obtained based on an elemental analysis of the sample, and the average content of SiCw in the composite was calculated to be about 2 wt%. The energy spectra of Si and Cu are shown in Figure 5g, and SiCw is randomly distributed on the surface of the composites.

In order to further observe the combination of SiCw and Cu on the surface of the coating, the surface of the coating was observed by high-rate scanning electron microscopy. As shown in Figure 6b, it can be observed that the surface of the coating is flat and the whiskers are evenly distributed. In addition, the Cu deposited on the coating grows along the direction of SiCw, which indicates that the TSV plating solution adopts a mode of growing from the bottom up [36]. This growth mode makes the bonding between Cu and whiskers stronger so that whiskers can give full play to their toughening effect in the face of external stress, can consume more fracture energy and is expected to further improve the mechanical properties of the material. In order to characterize the interfacial microstructure of Cu/SiCw composites, the composites were observed by TEM. As shown in Figure 6c, there are no defects at the interface, showing the typical Cu/SiCw/Cu sandwich structure, which is expected to enhance the performance of the composite.

#### 3.2.2. Cross-Sectional Morphology of TSV Composite Coating

**Figure 7:**
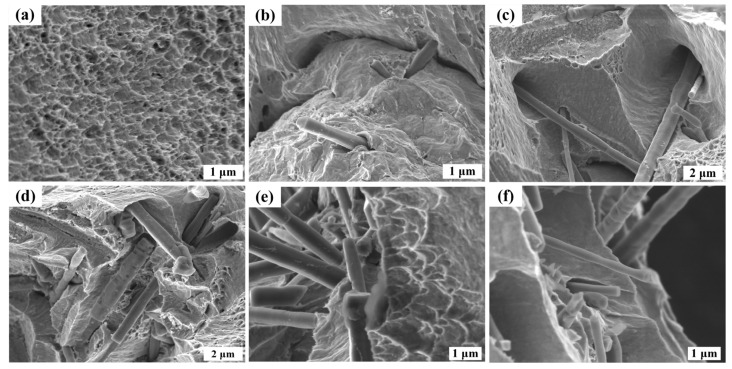
SEM images of the fracture surface of Cu/SiCw composites with different SiCw concentrations, (**a**) 0 g/L, (**b**) 0.2 g/L, (**c**) 0.5 g/L, (**d**) 0.8 g/L, (**e**) 1.2 g/L and (**f**) 1.5 g/L, in the plating solution.

In order to further verify whether the interior of the coating is consistent with the surface morphology, we characterized the cross-sectional morphology of the coating. SEM micrographs of the cross-sections of the fractured specimens with different SiCw fractions are shown in Figure 7. It is observed that the fracture surface exhibits uneven characteristics, which indicates that the introduction of whiskers changes the crack path. The cross-sectional morphology of copper is shown in Figure 7a, and a large number of dimples appear at the cross-section, which indicates that copper has good toughness. The concentration range of SiCw in the plating solution is 0.2 to 1.2 g/L. The fracture surface is shown in Figure 7b–e. It can be clearly observed on the fracture surface that the number of SiCw dispersed in the Cu matrix increases, and more and more whiskers are observed on the cross-section from the matrix, indicating that the fracture energy is consumed more. Especially when the concentration of SiCw in the plating solution is 1.2 g/L, a large number of dispersed whiskers can be observed on the cross-section of the coating, as shown in Figure 7e. In addition, a large number of dimples appear in the copper matrix, which indicates that the composite material still retains the toughness of the copper matrix under the toughening of the whisker; however, when the content of SiCw in the plating solution increases to 1.5 g/L, as shown in Figure 7f, SiCw becomes more and more uneven and agglomerates, which indicates that the whiskers do not improve the mechanical properties of the composites but weaken the characteristics of Cu itself, and the aggregated SiCw will lead to stress concentration, which is very unfavorable for SiCw toughening. Therefore, the dispersion of SiCw plays an important role and key influence on the mechanical properties of the composites.

**Figure 8:**
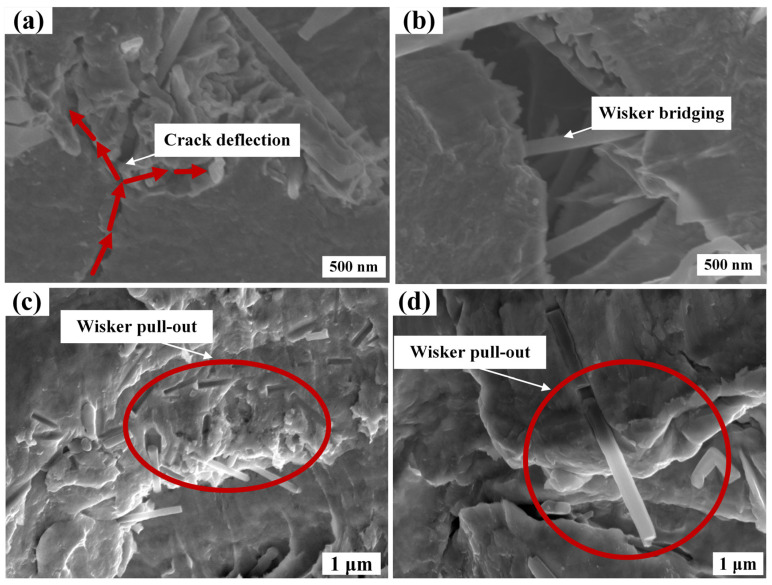
SEM images of Cu/SiCw composites near the fracture surface: (**a**) whisker crack deflection (red arrows indicate the direction of crack growth); (**b**) whisker bridging; (**c**) traces left by whisker pull-out; (**d**) whisker pull-out.

In order to further study the toughening mechanism of SiCw, the fracture surface of the Cu/SiCw composites was analyzed. The fracture surface is shown in Figure 8, which shows the toughening effect of the whiskers. As shown in Figure 8a, the presence of SiCw will lead to slight crack deflection. When the crack propagates to SiCw, due to the high modulus of the whisker, the crack tends to deflect around SiCw rather than directly penetrate SiCw, which prolongs the crack propagation path, consumes more energy through crack deflection and is expected to further improve the mechanical strength of the composite. The bridging toughening mechanism of the whiskers is shown in Figure 8b. When the whiskers are embedded in the matrix on both sides of the crack, further propagation of the crack is prevented, and the whiskers bear the fracture energy of the crack propagation, thereby improving the mechanical strength of the composites. The toughening mechanism of the whisker pull-out effect is shown in Figure 8c,d. It can be seen that traces are left when the whisker is pulled out of the matrix and the whiskers pulled out from the matrix. According to the shear lag theory, the shear stress is the interaction between SiCw and the matrix by chemical bonds. When the fracture energy makes a relative displacement of SiCw and the matrix reaches critical shear displacement, the chemical bond breaks, the whisker is pulled out and the fracture energy is consumed [37,38]. In summary, all SiCw toughening mechanisms are expected to improve the mechanical strength of composites by consuming more fracture energy.

## 4. Conclusions

In this paper, Cu/SiCw composites were prepared by composite electrodeposition. The effects of two different plating solutions on the microstructure of the composites at different SiCw concentrations were studied. The main conclusions are as follows:

1. The surface of a Cu/SiCw composite coating prepared with the Systek UVF 100 plating solution of the CuSO_4_ system is very flat and the whiskers are evenly distributed, but the combination of whiskers and Cu is not good and there are pores at the junction. When the whiskers at the cross-section of the coating are less, no pores are found at the cross-section. When the whiskers at the cross-section increase, pores appear in the Cu matrix. When the concentration of SiCw in the plating solution was 1.2 g/L, a more random distribution of whiskers was observed on the surface of the coating.
2. For the Cu/SiCw composite prepared with the TSV plating solution of the Cu(CH_3_SO_3_)_2_ system, the whiskers are evenly distributed on the surface of the flat coating, Cu grows along the whiskers and the whiskers are well combined with Cu. There is no porosity in the cross-section of the coating, and SiCw toughening mechanisms such as crack deflection, bridging and whisker pull-out are found at the cross-section. By analyzing the surface and cross-section of the composite coating, it is found that when the whisker concentration in the plating solution is 1.2 g/L, the whiskers observed on the surface of the coating are more and randomly distributed, which could enable SiCw to give full play to its toughening effect and is expected to further improve the mechanical properties of the material.

## Figures and Tables

**Table 1:** Composition of TSV electrolyte.

Composition of Bath	Values
Cu(CH_3_SO_3_)_2_	80~120 g/L
CH_3_SO_3_H	16~24 g/L
Cl^−^	0.04~0.06 g/L
DVF-B	5 mL/L
DVF-C	7 mL/L
DVF-D	5 mL/L
Applied density	Direct current

**Table 2:** Composition of Systek UVF 100 electrolyte.

Composition of Bath	Values
CuSO_4_·5H_2_O	210~270 g/L
H_2_SO_4_	30~60 g/L
Cl^−^	45~55 ppm
SYSTEK UVF 8470	10 mL/L
SYSTEK UVF 8471	1 mL/L
SYSTEK UVF 8472	20 mL/L
Applied density	Direct current

## Data Availability

The data are available from the authors on request.
